# Comparison between flipped classroom and lecture-based classroom in ophthalmology clerkship

**DOI:** 10.1080/10872981.2017.1395679

**Published:** 2017-11-02

**Authors:** Fen Tang, Chuan Chen, Yi Zhu, Chengguo Zuo, Yimin Zhong, Nan Wang, Lijun Zhou, Yuxian Zou, Dan Liang

**Affiliations:** ^a^ State Key Laboratory of Ophthalmology, Zhongshan Ophthalmic Center, Sun Yat-sen University, Guangzhou, People’s Republic of China; ^b^ Department of Molecular and Cellular Pharmacology, University of Miami Miller School of Medicine, Miami, FL, USA.

**Keywords:** Medical education, ophthalmology, clerkship, flipped classroom, lecture-based classroom

## Abstract

**Background**: In recent years, the flipped classroom method of teaching has received much attention in health sciences education. However, the application of flipped classrooms in ophthalmology education has not been well investigated.

**Objective**: The goal of this study was to investigate the effectiveness and acceptability of the flipped classroom approach to teaching ophthalmology at the clerkship level.

**Design**: Ninety-five fourth year medical students in an ophthalmology clerkship were randomly divided into two groups. An ocular trauma module was chosen for the content of this study. One group (FG (flipped group), n = 48) participated in flipped classroom instruction and was asked to watch a recorded lecture video and to read study materials before a face-to-face class meeting. They used the in-class time for discussion. The other group (TG (traditional group), n = 47) was assigned to traditional lecture-based instruction. These students attended a didactic lecture and completed assigned homework after the lecture. Feedback questionnaires were collected to compare students’ perspectives on the teaching approach they experienced and to evaluate students’ self-perceived competence and interest in ocular trauma. Pre- and post-tests were performed to assess student learning of the course materials.

**Results**: More students in the FG agreed that the classroom helped to promote their learning motivation, improve their understanding of the course materials, and enhance their communication skill and clinical thinking. However, students in the FG did not show a preference for this method of teaching, and also reported more burden and pressure than those from the TG. Students from the FG performed better on the post test over the ocular trauma-related questions when compared to those from the TG.

**Conclusions**: The flipped classroom approach shows promise in ophthalmology clerkship teaching. However, it has some drawbacks. Further evaluation and modifications are required before it can be widely accepted and implemented.

**Abbreviations** FG: Flipped classroom group; TG: Traditional lecture-based classroom group; TBL: Team-based learning; PBL: Problem-based learning; ZOC: Zhongshan Ophthalmic Center

## Introduction

The global trend in population aging calls for an increased number of well-trained ophthalmologists to provide eye care to the elderly, who commonly suffer from ophthalmic disorders, including cataract (and intraocular lens-related problems), age-related macular degeneration (AMD), and diabetes retinopathy [1–5]. Ophthalmic education is essential not only for the training of future ophthalmologists, but also for medical practitioners from other disciplines in general, as visual system dysfunction may provide clues for the diagnosis of systemic diseases [6,7]. Ophthalmology clerkship offers medical students a valuable opportunity to develop core competencies in ophthalmic clinical training, including patient care, medical knowledge, practice-based learning and improvement, interpersonal and communication skills, professionalism, and systems-based practice [8]. However, the constraints in time, teacher availability, funding, and resources allotted to ophthalmic education has gradually marginalized the ophthalmology clerkship in medical schools worldwide [1]. Lack of a solid foundation of ophthalmic knowledge and skills result in increased chances of misdiagnosis in daily clinical practice [9]. Additionally, limited understanding of ophthalmology will demotivate medical students to pursue ophthalmology as their subspecialty after graduation [10]. Subsequently, there exists a growing concern for the general quality of ophthalmic education which has created a need for reform of current teaching methods to better prepare students for future careers in medical practice.

The most effective approach to improve teaching efficiency is to promote active learning, which requires students to actively engage with learning materials, participate in the class, and collaborate with other classmates [11–13]. Our group and others have studied the implementation of several teaching approaches in ophthalmology clerkships that promote student-centered learning. For example, both teachers and students express positive feelings about the effectiveness of team-based learning in ophthalmology [14]. More recently, we demonstrated that team-based learning (TBL) can significantly improve students’ performance and engagement in an ophthalmology clerkship [15]. Additionally, problem-based learning (PBL), which requires students to work on ‘real-life’ scenarios, can stimulate student’s interest in ophthalmology and improve their clinical skills, including collecting medical history and performing eye examinations [16]. These teaching methods mobilize student enthusiasm for learning ophthalmology and promote students’ engagement, interaction, and cooperation in learning.

Recently, the flipped classroom approach has received much attention in medical education [17]. The flipped classroom requires students to obtain background knowledge through homework prior to a face-to face class meeting, and reserves precious in-class time for applying knowledge to solve real clinical problems through discussion facilitated by faculty [18,19]. This is the opposite of the traditional lecture-based classroom, in which students attend didactic lectures where they obtain knowledge passively from the instructor, then study the content and complete assignments after class. Previous studies have shown that the flipped classroom can provide students more flexibility for self-paced learning, help to promote content retention, and promote students’ interest in learning [18,20]. However, the overall effectiveness of the flipped classroom approach in medical education is still being debated. For example, Whillier et al. showed that the flipped classroom did not improve students’ performance nor satisfaction in learning neuroanatomy [21], suggesting that the flipped classroom may not be suitable for learning abstract and memorization-heavy concepts. Therefore, it is important to evaluate the effectiveness of the flipped classroom each time that it is applied to a new setting.

A large body of recent literature has reported the application of flipped classroom in health sciences education, including nursing, pharmacology, physiology, radiology, epidemiology, and stomatology [17,22–30]. More recently, the flipped classroom approach has been extended to medical clerkship teaching with encouraging results, such as clerkships in emergency medicine and surgery [22,31–33]. However, the application of the flipped classroom in ophthalmology education is less well studied. Previous studies from our group took the first step in applying the flipped classroom approach to clerkship teaching of glaucoma and ocular trauma, and showed that the flipped classroom was welcomed by both students and teachers [34,35]. More importantly, the flipped classroom helped students to develop skills in problem-solving, creative and critical thinking, and team work [34]. However, this pilot study had a relatively small sample size, and was not designed to compare students’ perspectives of both flipped and traditional lecture-based classrooms. To further evaluate the effectiveness and acceptability of the flipped classroom in ophthalmology clerkship teaching, we performed the current randomized control study involving 95 medical students. We compared the students’ workload, interest, and overall performance between the flipped classroom and traditional lecture-based classroom. The purpose of this paper is to provide guidance for instructors who are considering using the flipped classroom approach in ophthalmology education.

## Materials and methods

### Subjects and study design

Ninety-five fourth year students majoring in clinical medicine at the medical school of Sun Yat-sen University were enrolled in the ophthalmology clerkship at Zhongshan Ophthalmic Center (ZOC). They had attended all the ophthalmology lectures provided in Sun Yat-sen University by the same instructors. These participants were randomly allocated into either the flipped classroom group (FG, n = 48) or the traditional lecture-based classroom group (TG, n = 47). All students were unaware of their group assignments before the clerkship. Both classroom groups had one professor and five teaching assistants, who were residents in ZOC. Informed consent was obtained from all subjects, and the research was approved by the Institutional Review Board of Zhongshan Ophthalmic Center (IRB-ZOC-SYSU).

### Curriculum description

**Figure 1.**
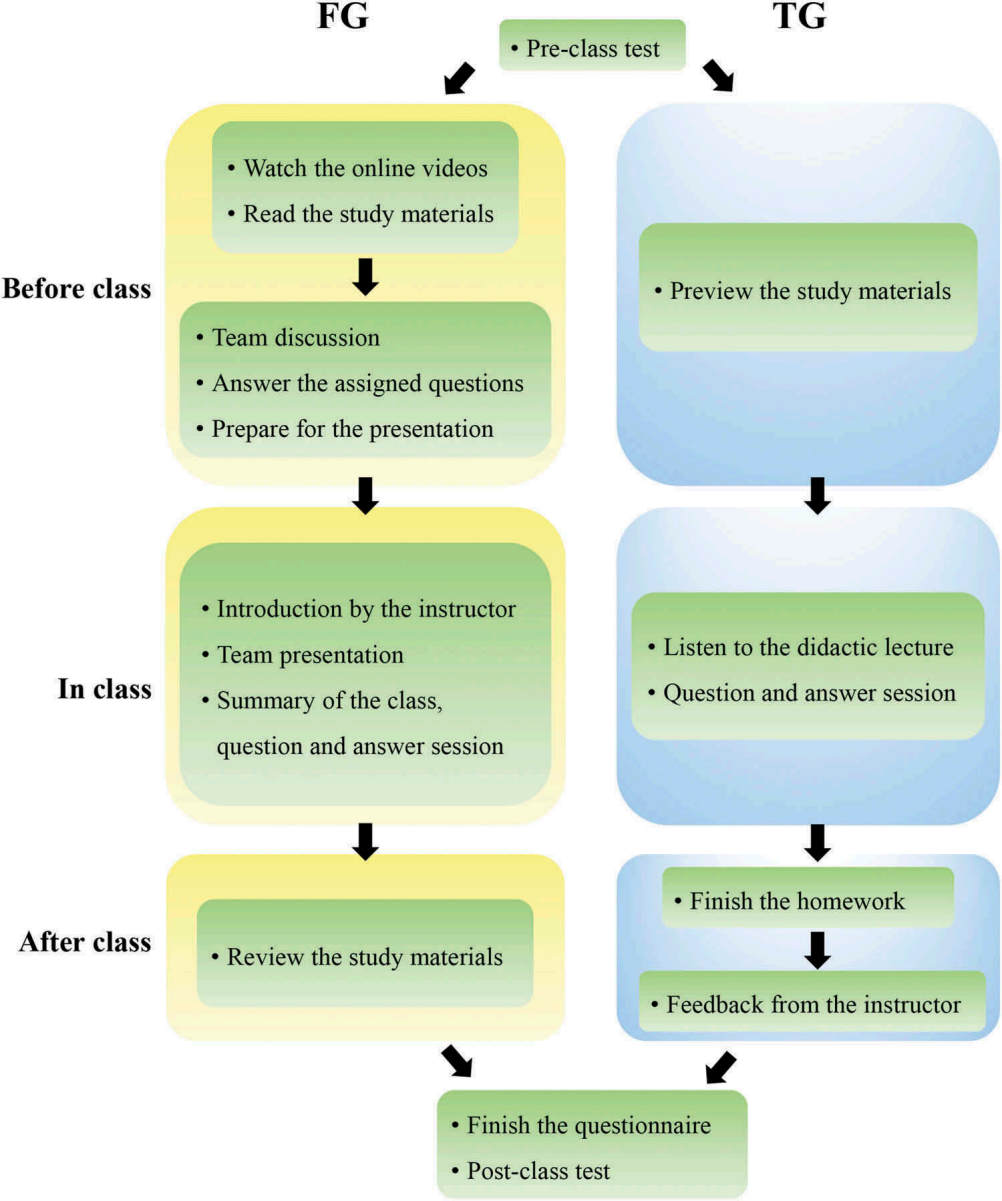
Flow diagram illustrating the flipped classroom and traditional lecture-based classroom models. FG: flipped classroom group, TG: traditional lecture-based classroom group.

The full curriculum of ophthalmology clerkship (50 hours) was divided into six clinical modules, including eye examinations, corneal diseases, cataract, glaucoma, retinal diseases, and ocular trauma. According to the feedback from our previous study, teachers in ZOC agreed that ocular trauma is a more suitable topic for the implementation of the flipped classroom approach compared to other topics. Consistently, students had evaluated the flipped ocular trauma classroom more positively than the flipped glaucoma classroom [34]. Ocular trauma cases usually cover multiple eyeball injuries, which requires students to have a comprehensive grasp of knowledge about ophthalmic diseases, including endophthalmitis, glaucoma, cataract, iris concussion, vitreous hemorrhage, and retinal detachment. Additionally, the clinical signs and symptoms in ocular trauma are easier for students to observe and understand. Therefore, we chose the ocular trauma module to apply the flipped classroom approach for this study. The chronology of the flipped classroom and traditional lecture-based classroom is summarized in Figure 1.

To encourage collaboration in the flipped classroom group, students were further organized into small teams, with four students per team. Before the classroom session, the professor prepared the course materials, including a recorded lecture video, supplementary study materials, and several relevant questions. Representative questions included: (1) How to choose the operation time for traumatic cataract? (2) What is the pathogenesis of traumatic glaucoma? (3) Can we use immunosuppressive agents to prevent sympathetic ophthalmia? Each team needed to watch the lecture video, discuss the questions, and prepare a PowerPoint presentation for in-class discussion. The flipped classroom session started with a brief introduction of the topic and class agenda by the professor. After that, a student representative from each team made a ten-minute presentation to review the main points from the lecture. The student also presented the unsolved questions from the team for in-class discussion. Then, each team proposed their answers to the questions, and discussed the answers for about 20 minutes. For some particularly challenging questions, students were encouraged to consult the literature and with teaching assistants. In the end, the professor summarized for the class, going over the tough questions raised during discussion. The students were encouraged to review the study materials after class.

The traditional lecture-based classroom followed the same procedure as we described in our previous study [34]. Briefly, the students attended a two-hour didactic lecture followed by a 30-minute question-and-answer session. After the class, the instructor assigned homework involving the same questions discussed in the flipped classroom. The students were asked to complete and hand in the homework within one week. The instructor gave feedback to all the students, posted the answers to the questions online, and helped students to work through particularly challenging questions at the students’ request. As a control, the students assigned in the lecture-based classroom group had the same access to the recorded lecture video and supplementary study materials as those in the flipped classroom group, and they were also encouraged to preview the study materials before attending the lecture.

### Data collection and statistical analysis

Before the ophthalmology clerkship, all students were asked to complete a pretest of 50 questions. After the classroom, students from both groups were required to complete an anonymous questionnaire to evaluate their perspectives of the module (nine items covering both positive and negative aspects). Additionally, after completing the module, students were asked to self-evaluate their competence and interest in ocular trauma. Students’ perspectives and self-evaluations were quantified using a three-point Likert-type scale (−1, disagree; 0, neutral; 1, agree). The questionnaire was modified from Paul Ramsden’s Course Experience Questionnaire and Biggs’ Study Process questionnaire with verified reliability and validity [36,37]. Moreover, students needed to report how many hours they had spent on their preparation for the lesson. To evaluate students’ understanding of the course material, a post-test was conducted after the students had finished all the modules in the clerkship. The post-test was composed of multiple choice questions, and each question had the same weight. We calculated the total scores of ocular trauma-related questions and non-ocular trauma-related questions for each student.

All the questionnaire data were analyzed using the Mann-Whitney U test. The hours spent on class preparation were analyzed by independent t-test. The level of students’ interest in ocular trauma was quantified as follows: 1, not interested; 2, somewhat interested; 3, very interested, and we compared the two groups using the Mann-Whitney U test [38]. The pretest and post-test scores were compared between the two groups by an independent samples t-test. All preliminary statistical analyses were performed using the SPSS 20.0 version (Chicago, USA). Alpha was set at 0.05, and p-values of less than 0.05 were considered statistically significant. Cohen’s D effect sizes were calculated using the Psychometrica online effect size calculators [39]. Cohen’s D effect sizes are commonly interpreted as 0.0–0.1 = no effect; 0.2–0.4 = small effect; 0.5–0.7 = moderate effect; and 0.8–1.0 = large effect.

## Results

A total of 95 students were enrolled in the study, including 48 students assigned to the flipped classroom group and 47 students assigned into the traditional lecture-based classroom group. The gender ratio and ages for the two groups were comparable (Table 1). The class attendance rates of both groups were 100%. All students in the flipped classroom group watched the online lecture video and read the supplementary study materials assigned by the instructor. All students in the traditional lecture-based classroom group completed and submitted the homework to the instructor on time. The response rates for the questionnaires were 100% for both groups. However, among the questionnaires received, some had all questions scored ‘-1’ or ‘1’. For a more objective analysis, we eliminated these data from statistical analysis. A total number of 76 reliable questionnaires were analyzed, with 41 from the flipped classroom group (85.4%) and 35 from the traditional lecture-based classroom group (74.5%).

**Table 1.** Demographic information of medical students who participated in an ophthalmology clerkship study.

	FG	TG	Statistics	df	P value
Number of students	48	47			
Gender					0.752^a^
Male	25 (52.1%)	26 (55.3%)	*X*^2^ = 0.1 (df = 1)	1	
Female	23 (47.9)	21 (44.7%)			
Age (years old)	22.3 ± 0.6	22.6 ± 0.4	t = −1.23 (df = 93)	93	0.223^b^

**Figure 2.**
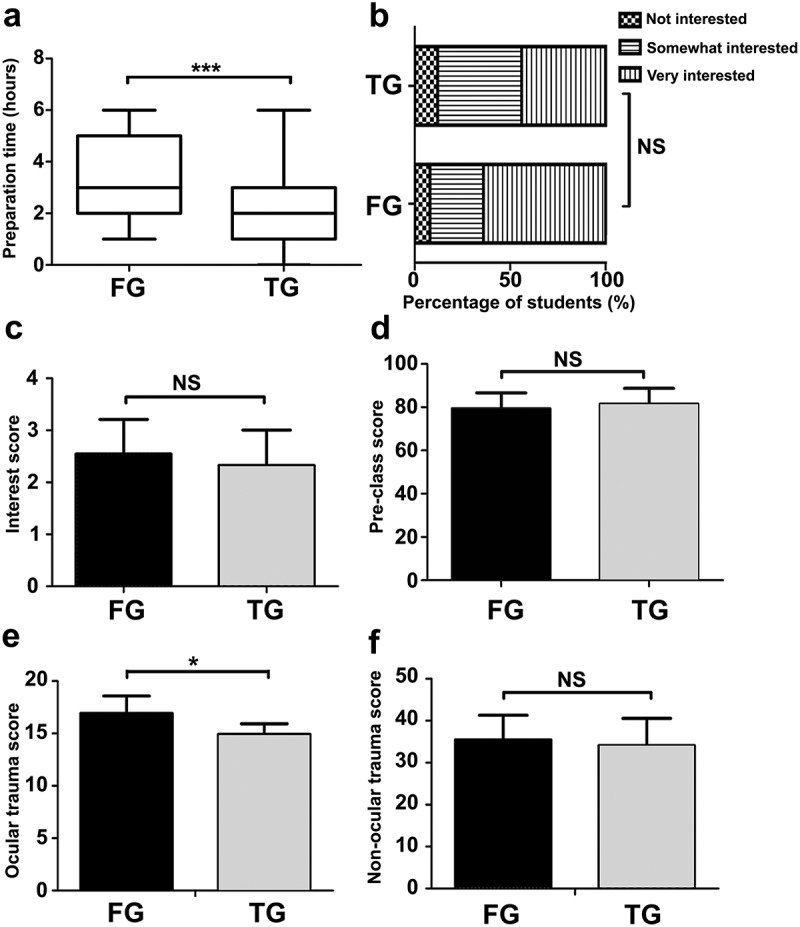
Feedback from students taking the flipped classroom compared to those taking the An iAraditional lecture-based classroom. (a) Box plot indicating the preparation time for the class between the two groups (hours). An independent samples t test was performed to compare the differences between the two groups. t = 3.651 (df = 74), Effect size = 0.91, ***P ≤ 0.001. (b) Stacked column charts indicating the percentage of students interested in ocular trauma after taking the flipped classroom and traditional lecture-based classroom, respectively. A Mann-Whitney U test was used to compare the data from the two groups. (c) The level of students’ interest in ocular trauma was quantified as follows: 1, not interested; 2, somewhat interested; 3, very interested. A Mann-Whitney U test was performed to compare the differences between the two groups. U = 570.0 (Z = −1.727), Effect size = 0.36, P > 0.05. (d) Comparison of students’ test scores before the classroom. Data were presented as mean ± S.D. An Independent samples t test was used to compare the differences between the two groups. t = −1.495 (df = 74), P = 0.14, Effect size = 0.45. (e and f) Comparison of students’ test scores after the classroom. The ocular trauma-related questions (e) and non-ocular trauma-related questions (f) were scored, respectively. An Independent samples t test was used to compare the differences between the two groups. Data were presented as mean ± S.D. In ocular trauma-related questions, t = 2.64 (df = 74), *P = 0.01, Effect size = 1.44; In non-ocular trauma-related questions, t = 1.24 (df = 74), P = 0.22, Effect size = 0.21. NS: not significant, FG: flipped classroom group, TG: traditional lecture-based classroom group.

Table 2 compares students’ perspectives on the flipped and traditional lecture-based classrooms. More students in the flipped classroom group agreed that the clerkship could help to improve their motivation in learning ocular trauma (P = 0.012), understand the course material (P = 0.029), and prepare for the examination (P = 0.001). However, flipped-classroom students did not show more satisfaction (P = 0.610) or preference (P = 0.253) for the flipped classroom method. Instead, the students in the flipped classroom group felt more ‘burden and pressure’ compared to the students in the lecture-based classroom group (P = 0.007). This finding may be explained by the fact that students in the flipped classroom group spent significantly more time preparing for class than those from the lecture-based classroom group (3.52 ± 1.42 hours vs. 2.24 ± 1.41 hours, P < 0.001, Effect size = 0.91, Figure 2(a)). These results suggest that flipped classroom approach can enhance students’ sense of active participation, while at the same time increase their workload.

**Table 2.** Comparison of students’ perspectives between flipped classroom and traditional lecture-based classroom in ocular trauma clerkship.

Items	Group	Disagree	Neutral	Agree	Statistics	P value^a^	Effect size^b^
The course improves my learning motivation.	FG	0 (0%)	12 (29.2%)	29 (70.8%)	U = 511.5	0.012*	0.60
	TG	1 (2.8%)	19 (54.3%)	15 (42.9%)			
The course is helpful for understanding the course material.	FG	0 (0%)	22 (48.8%)	21 (51.2%)	U = 536.5	0.029*	0.51
	TG	3 (8.6%)	23 (65.7%)	9 (25.7%)			
The course is helpful for the final examination.	FG	1 (2.4%)	20 (48.8%)	20 (48.8%)	U = 443	0.001**	0.70
	TG	4 (11.4%)	26 (74.3%)	5 (14.3%)			
I am satisfied with the course.	FG	0 (0%)	18 (43.9%)	23 (56.1%)	U = 675	0.610	0.10
	TG	1 (2.9%)	16 (45.7%)	18 (51.4%)			
I like this teaching method.	FG	0 (0%)	18 (43.9%)	23 (56.1%)	U = 622.5	0.253	0.23
	TG	0 (0%)	20 (57.1%)	15 (42.9%)			
I would like this teaching method to be applied in the future ophthalmology curriculum.	FG	2 (4.9%)	21 (51.2%)	18 (43.9%)	U = 638.5	0.351	0.19
	TG	1 (2.9%)	15 (42.8%)	19 (54.3%)			
This course gives me too much burden and pressure	FG	8 (19.5%)	23 (56.1%)	10 (24.4%)	U = 483.0	0.007**	0.58
	TG	15 (42.9%)	18 (51.4%)	2 (5.7%)			
This course occupies too much of my spare time.	FG	9 (22.0%)	24 (58.5%)	8 (19.5%)	U = 601.5	0.169	0.28
	TG	11 (31.4%)	21 (60.0%)	3 (8.6%)			
I need to spend a lot of energy on this course.	FG	16 (39.9%)	25 (60.1%)	0 (0%)	U = 669.5	0.559	0.12
	TG	16 (45.7%)	19 (54.3%)	0 (14.3%)			

Table 3 compares students’ self-perceived competence after taking the flipped classroom and lecture-based classroom. More students in the flipped classroom group agreed that the clerkship improved their communication skills (P = 0.037) and promoted clinical thinking (P = 0.049). However, in terms of ‘knowledge acquisition,’ ‘presentations in public,’ and ‘scientific thinking,’ responses from the two groups did not show a difference. Additionally, 92% students in the flipped classroom group said that they were ‘somewhat interested’ or ‘very interested’ in ocular trauma after the clerkship, which was only slightly higher than students in the traditional lecture-based classroom group (88%) (Figure 2(b)). Overall interest scores between the two groups were not considered significantly different (P > 0.05) (Figure 2(c)). These findings indicate that although the flipped classroom method in ocular trauma clerkship teaching may improve students’ communication skills and promote clinical thinking, it does not increase their interest in ocular trauma.

**Table 3.** Comparison of students’ self-perceived competence after flipped classroom and traditional lecture-based classroom methods in ocular trauma clerkship.

Items	Group	Disagree	Neutral	Agree	Statistics	P value^a^	Effect size^b^
The course improves my communication ability.	FG	1 (2.4%)	20 (48.8%)	20 (48.8%)	U = 544	0.037*	0.42
	TG	2 (5.7%)	24 (68.6%)	9 (25.7%)	(Z = −2.087)		
The course improves my clinical thinking ability.	FG	1 (2.4%)	11 (26.8%)	29 (70.7%)	U = 555.5	0.049*	0.40
	TG	2 (5.7%)	16 (45.7%)	17 (48.6%)	(Z = −1.971)		
The course improves my ability to acquire knowledge.	FG	0 (0%)	12 (29.3%)	29 (70.7%)	U = 654.5	0.446	0.15
	TG	1 (2.9%)	14 (40%)	20 (57.1%)	(Z = −0.762)		
The course improves my ability to give presentations and express my opinions.	FG	0 (0%)	21 (51.2%)	20 (48.8%)	U = 705.5	0.886	0.03
	TG	0 (0%)	22 (62.9%)	13 (37.1%)	(Z = −0.143)		
The course improves my ability in scientific thinking.	FG	2 (4.9%)	22 (53.6%)	17 (41.5%)	U = 660.5	0.500	0.14
	TG	1 (2.8%)	17 (48.6%)	17 (48.6%)	(Z = −0.675)

There were no differences between the flipped classroom group and traditional lecture-based classroom with regard to their pretest score (78.55 ± 7.09 vs. 81.74 ± 7.08, P = 0.14, Effect size = 0.45), indicating that the baseline knowledge of ophthalmology between the two groups was comparable (Figure 2(d)). The post-test showed that students in the flipped classroom group had significantly higher scores in ocular trauma questions than those from the traditional lecture-based classroom (16.91 ± 1.67 vs. 14.92 ± 1.01, P = 0.01, Effect size = 1.44, Figure 2(e)). However, the non-ocular trauma scores between the two groups were still comparable (35.43 ± 5.88 vs. 34.12 ± 6.44, P = 0.22, Effect size = 0.21, Figure 2(f)).

## Discussion

This study was a further investigation into the effectiveness and suitability of the flipped classroom model in an ophthalmology clinical clerkship, which was developed based on our previous pilot study [34]. This study was an improvement over the pilot in that: (1) we optimized the flipped classroom approach in ocular trauma teaching due to our previous finding that both students and teachers considered the ocular trauma subject more suitable for flipped classroom [34]; (2) this study compared the students’ perspectives between flipped classroom and a traditional lecture-based classroom in ophthalmology clerkship; and (3) this study had a larger sample size. Overall, students in the flipped classroom group felt more motivated for learning when compared to those in the traditional lecture-based classroom group. Additionally, students considered the flipped classroom teaching approach to be more helpful for learning the course material and for exam preparation. Moreover, students in the flipped classroom group felt better improvement in their communication and critical thinking skills. Lastly, the post-test revealed that the flipped classroom students performed better on the content covered by the module on ocular trauma (Figure 2(e)). Notably, the baseline performance before ophthalmology clerkship of both groups was comparable as indicated by the pretest (Figure 2(d)). The performance on the post-test was comparable for both groups on the non-ocular trauma related questions (Figure 2(f)). These findings should be particularly encouraging to educators to consider using the flipped classroom approach since we have shown that it is very effective in helping students master and apply ophthalmology knowledge.

Multiple factors may contribute to the effectiveness of the flipped classroom method for use with ophthalmology clerkship. First, the flipped classroom approach offers personalized study. Students in the flipped classroom group have more freedom and flexibility of self-paced learning [34], giving students an opportunity to use their time more efficiently. Second, the flipped classroom approach offers group study. Compared to the traditional lecture-based classroom in which there is only teacher-student interaction, the flipped classroom encourages not only teacher-student interaction but also student-student interaction. Studying as a group may contribute to improving individual student’s mastery of medical knowledge. Third, the flipped classroom approach emphasizes the output of knowledge from students. The traditional lecture-based classroom focuses on how much knowledge can be absorbed in class by the students through reading and listening (input); however, in the flipped classroom, students are encouraged to verbalize what they learn and to exchange ideas through discussion or debate (output). The output process is further encouraged by the professor and teaching assistants in the flipped classroom. Compared to the traditional lecture-based classroom where medical students have only an average attention span of 10–20 minutes at the beginning of the lecture [40], the flipped classroom approach engages students longer, which may aid in knowledge retention. Consistent with our findings, previous studies have shown that the flipped classroom approach improves students’ performance [22,24,33].

Interestingly, although students in the flipped classroom performed better on the post-test, they gave negative feedback about the burden and pressure of preparing for the flipped classroom (Table 2). The flipped classroom model emphasizes student-oriented, active learning. This is more motivating but can be challenging to students who are overwhelmed by the quantity of learning material covered in medical school. Because the flipped classroom requires additional time for self-learning, problem solving, and preparation for in-class presentation and discussion, it was considered an unnecessary burden by strategic learners who focus more on their immediate learning needs for tests, and do not consider their learning needs for the future. Alternatively, this negative feedback might be attributed to the learner’s reluctance to change when a new teaching method is introduced. The burden and pressure may compromise the satisfaction that students felt as our questionnaire showed no difference in the satisfaction with the course or the teaching approach between the two groups. Moreover, we found that students showed no preference for the flipped classroom in future ophthalmology clerkship (Table 2). As educators, the default reasoning might be ‘no pain, no gain’, where more engagement of students generates better performance. However, in real practice, educators may need to strike a balance between different teaching methods. For example, students may be involved in both an ophthalmology and internal medicine clerkship during the same period of time. Too much burden from one subject may squeeze the time for the others. Therefore, the benefits of the flipped classroom may be less valuable if the students feel overwhelmed. Consistent with this idea, we found that students in the flipped classroom group do not show more interest in ophthalmology after the clerkship (Figure 2(c)).

A few points need to be highlighted for medical educators who may be considering the application of flipped classroom in teaching. First, consider whether the medical subject is suitable for flipped classroom instruction We found that the flipped classroom model is more favorable for content material that is more concrete and less abstract. For example, previous studies show that the flipped classroom approach used in glaucoma [34] and neuroanatomy [21] does not increase students’ exam scores, where both courses are too abstract and memorization-heavy. Second, consider how much pre-class workload is suitable for students using the flipped classroom approach. The pre-class workload may include a pre-recorded video lecture, assigned reading materials, and preparation for in-class presentations and discussion. There is no conclusive suggestion yet from previous literature on what the pre-class workload should be; however, our current study indicated that the average pre-class preparation time of 3.5 hours triggered student complaints about a workload burden. Future studies may be warranted in order to optimize the time dedicated to the flipped classroom approach. Third, an updated evaluation system may be needed to better evaluate the effectiveness of the flipped classroom [34]. Core competencies required of doctors in training, such as teamwork and communication, may not be sufficiently measured by the multiple-choice questions-based exam. Accordingly, the added value of the flipped classroom may be underestimated due to the inadequacy of the traditional exam to measure competencies other than knowledge achievement. Future educators may need to evolve the student evaluation system accordingly when they implement the flipped classroom approach.

### Study limitations

Several limitations need to be considered. First, some evaluation of the flipped classroom model is based on self-assessment from the participating students, such as communication skills and clinical thinking. As discussed above, an updated evaluation system which includes measures of core competencies other than knowledge acquisition will be warranted to better evaluate the effectiveness of flipped classroom. Second, we investigated only the pre-class time that students spent on both classroom methods; however, we did not collect the after-class time that students took. Students in the traditional lecture-based classroom group may spend more time after class to review the lecture and do the homework assignments, compared to the flipped classroom participants whose work was done before and during class time.

## Conclusion

The flipped classroom approach could be a better option over the traditional lecture-based classroom in the teaching of the ocular trauma module during ophthalmology clerkship. Flipped classroom stimulates students’ learning motivation, improves their performance in the final exam, and may help to enhance clinical thinking and communication skills. The flipped classroom approach needs to be further optimized in terms of specific subjects, students’ workload, as well as the evaluation system of students’ performance.
