# Erratum

**DOI:** 10.1080/10872981.2017.1406198

**Published:** 2017-11-25

Tang F, Chen C, Zhu Y, et al. Comparison between flipped classroom and lecture-based classroom in ophthalmology clerkship. Med Educ Online. 2017;22(1):1395679.

**Table 1.** Demographic information of medical students who participated in an ophthalmology clerkship study.

	FG	TG	Statistics	df	P value
Number of students	48	47			
Gender					0.752^a^
Male	25 (52.1%)	26 (55.3%)	*X*^2^ =0.1 (df =1)	1	
Female	23 (47.9)	21 (44.7%)			
Age (years old)	22.3 ± 0.6	22.6 ± 0.4	t=–1.23 (df=93)	93	0.223^b^

FG: flipped classroom group, TG: traditional lecture-based classroom group, df = degrees of freedom ^a^The two groups were compared using the Pearson Chi-Square test. ^b^The two groups were compared using Independent samples t test.

**Table 2.** Comparison of students’ perspectives between flipped classroom and traditional lecture-based classroom in ocular trauma clerkship

Items	Group	Disagree	Neutral	Agree	Statistics	P value^a^	Effect size^b^
The course improves my learning motivation.	FG	0 (0%)	12 (29.2%)	29 (70.8%)	U=511.5	0.012*	0.60
	TG	1 (2.8%)	19 (54.3%)	15 (42.9%)			
The course is helpful for understanding the course material.	FG	0 (0%)	22 (48.8%)	21 (51.2%)	U=536.5	0.029*	0.51
	TG	3 (8.6%)	23 (65.7%)	9 (25.7%)			
The course is helpful for the final examination.	FG	1 (2.4%)	20 (48.8%)	20 (48.8%)	U=443	0.001**	0.70
	TG	4 (11.4%)	26 (74.3%)	5 (14.3%)			
I am satisfied with the course.	FG	0 (0%)	18 (43.9%)	23 (56.1%)	U=675	0.610	0.10
	TG	1 (2.9%)	16 (45.7%)	18 (51.4%)			
I like this teaching method.	FG	0 (0%)	18 (43.9%)	23 (56.1%)	U=622.5	0.253	0.23
	TG	0 (0%)	20 (57.1%)	15 (42.9%)			
I would like this teaching method to be applied in the future ophthalmology curriculum.	FG	1 (2.9%)	21 (51.2%))	18 (43.9%)	U=638.5	0.351	0.19
	TG	1 (2.9%)	15 (42.8%)	19 (54.3%)			
This course gives me too much burden and pressure.	FG	8 (19.5%)	23 (56.1%)	10 (24.4%)	U=483.0	0.007**	0.58
	TG	15 (42.9%)	18 (51.4%)	2 (5.7%)			
This course occupies too much of my spare time.	FG	9 (22.0%)	24 (58.5%)	8 (19.5%)	U=601.5	0.169	0.28
	TG	11 (31.4%)	21 (60.0%)	3 (8.6 %)			
I need to spend a lot of energy on this course.	FG	16 (39.9%)	25 (60.1%)	0 (0%)	U=669.5	0.559	0.12
	TG	16 (45.7%)	19 (54.3%)	0 (14.3%)			

FG: flipped classroom group, TG: traditional lecture-based classroom group

Students’ answers to the survey questions were quantified using a three-point Likert scale (-1, disagree; 0, neutral; 1, agree). Data presented indicate the number (percentage) of students that chose the answer.

^a^The two groups were compared using the Mann-Whitney U test. *P<0.05, **P<0.01

^b^Cohen’s D effect sizes were calculated with the Effect size calculator for non-parametric tests (40)

**Table 3.** Comparison of students’ self-perceived competence after flipped classroom methods and traditional lecture-based classroom in ocular trauma clerkship

Items	Group	Disagree	Neutral	Agree	Statistics	P value^a^	Effect size^b^
The course improves my communication ability.	FG	1 (2.4%)	20 (48.8%)	20 (48.8%)	U=544	0.037*	0.42
	TG	2 (5.7%)	24 (68.6%)	9 (25.7%)	(Z=-2.087)		
The course improves my clinical thinking ability.	FG	1 (2.4%)	11 (26.8%)	29 (70.7%)	U=555.5	0.049*	0.40
	TG	2 (5.7%)	16 (45.7%)	17 (48.6%)	(Z=-1.971)		
The course improves my ability to acquire knowledge.	FG	0 (0%)	12 (29.3%)	29 (70.7%)	U=654.5	0.446	0.15
	TG	1 (2.9%)	14 (40%)	20 (57.1%)	(Z=-0.762)		
The course improves my ability to give presentations and express my opinions.	FG	0 (0%)	21 (51.2%)	20 (48.8%)	U=705.5	0.886	0.03
	TG	0 (0%)	22 (62.9%)	13 (37.1%)	(Z=-0.143)		
The course improves my ability in scientific thinking.	FG	2 (4.9%)	22 (53.6%)	17 (41.5%)	U=660.5	0.500	0.14
	TG	1 (2.8%)	17 (48.6%)	17 (48.6%)	(Z=-0.675)		

FG: flipped classroom group, TG: traditional lecture-based classroom group.

Students’ answers to the survey questions were quantified using a three-point Likert scale (-1, disagree; 0, neutral; 1, agree). Data presented indicate the number (percentage) of students who chose the answer.

^a^The two groups were compared using the Mann-Whitney U test. *P<0.05.

^b^Cohen’s D effect sizes were calculated with the Effect size calculator for non-parametric tests (40)

https://doi.org/10.1080/10872981.2017.1395679

When the above article was first published online, the tables were published without footnotes. This has now been corrected in the online version as below.

